# Validation of hybrid angular spectrum acoustic and thermal modelling in phantoms

**DOI:** 10.1080/02656736.2018.1513168

**Published:** 2018-10-15

**Authors:** Sara L. Johnson, Douglas A. Christensen, Christopher R. Dillon, Allison Payne

**Affiliations:** a Department of Bioengineering, University of Utah, Salt Lake City, UT, USA; b Department of Computer and Electrical Engineering, University of Utah, Salt Lake City, UT, USA; c Department of Radiology and Imaging Sciences, University of Utah, Salt Lake City, UT, USA

**Keywords:** Treatment planning, modeling, validation, high intensity focused ultrasound, thermal ablation

## Abstract

In focused ultrasound (FUS) thermal ablation of diseased tissue, acoustic beam and thermal simulations enable treatment planning and optimization. In this study, a treatment-planning methodology that uses the hybrid angular spectrum (HAS) method and the Pennes’ bioheat equation (PBHE) is experimentally validated in homogeneous tissue-mimicking phantoms. Simulated three-dimensional temperature profiles are compared to volumetric MR thermometry imaging (MRTI) of FUS sonications in the phantoms, whose acoustic and thermal properties are independently measured. Additionally, Monte Carlo (MC) uncertainty analysis is performed to quantify the effect of tissue property uncertainties on simulation results. The mean error between simulated and experimental spatiotemporal peak temperature rise was +0.33°C (+6.9%). Despite this error, the experimental temperature rise fell within the expected uncertainty of the simulation, as determined by the MC analysis. The average errors of the simulated transverse and longitudinal full width half maximum (FWHM) of the profiles were –1.9% and 7.5%, respectively. A linear regression and local sensitivity analysis revealed that simulated temperature amplitude is more sensitive to uncertainties in simulation inputs than in the profile width and shape. Acoustic power, acoustic attenuation and thermal conductivity had the greatest impact on peak temperature rise uncertainty; thermal conductivity and volumetric heat capacity had the greatest impact on FWHM uncertainty. This study validates that using the HAS and PBHE method can adequately predict temperature profiles from single sonications in homogeneous media. Further, it informs the need to accurately measure or predict patient-specific properties for improved treatment planning of ablative FUS surgeries.

## Introduction

Focused ultrasound (FUS) thermal ablation is a non-invasive localized surgical technique that has been used to treat a variety of pathologies in tissues such as prostate, brain, breast, bone, and benign and malignant tumors [1–4]. Thermal ablation parameters, such as sonication power and duration, are prescribed in order to achieve thermal dose accumulation in the targeted tissue. An accumulation of 240 cumulative equivalent minutes at 43°C is often clinically used to correlate with thermally necrotized tissue [5]. FUS ablations can be simulated prior to treatment using patient-specific models in order to determine the required FUS sonication power levels, spacing, and duration to achieve the thermal dose for the desired clinical outcome. Treatment planning through acoustic and thermal simulation has been shown to increase the success rate of treatments [6,7], reduce damage to healthy tissues [8,9], and shorten treatment times [10].

Both acoustic and thermal simulation techniques are required for FUS treatment planning. Acoustic simulations model the pressure field and power deposition Q (W/m^3^) of the FUS beam while thermal simulations model the resulting temperature rise used to calculate the thermal dose volume. Several approaches to modeling FUS acoustic pressure and Q patterns in tissues have been developed. Finite-difference approaches to the Khoklov-Zabolotskaya-Kuznetsov (KZK) [11,12] and Westervelt equations [13] for modeling non-linear acoustic propagation, or the Helmholtz equation [14–16] for linear propagation are commonly implemented, but are computationally expensive for clinical use. Numerous studies have improved the computation time of these models using methods such as the boundary element method, [14,17,18] or improving upon the FDTD approach with pseudo-spectral k-space methods, [19,20] or by applying approximations of temporal derivatives [21]. Although considerable improvements to computation time were achieved, parallel computing was often implemented [19] and computation times still ranged from several minutes to days [18,21,22].

The Helmholtz equation describing linear propagation can also be solved for quasi-steady-state conditions using the angular spectrum method, which takes advantage of the fast Fourier transform to calculate wave propagation in the frequency domain in order to decrease computation time [13,23], but its implementation has previously been limited to homogeneous models. The hybrid angular spectrum (HAS) method, developed by Vyas et al. [23], extends the angular spectrum method to the heterogeneous case and provides a computationally fast alternative to full-wave models for treatment planning purposes. Although the current HAS method does not model the effects of scattering and nonlinear propagation, its computational speed is attractive for integrative use with clinical treatment planning and guiding software. HAS was shown to correlate well with the FDTD approach of the Westervelt equation for propagation of a phased-array FUS transducer source through a 3 D model of heterogeneous tissue [23]. An additional comparison between HAS and k-Wave [20] simulations of a 3 D pressure pattern for a single sonication through a heterogeneous breast model including skin, fat, and fibroglandular tissue resulted in a normalized root-mean-squared difference of 2.9%, with HAS showing more than two orders of magnitude improvement in computation time. However, the accuracy of HAS for modeling the power deposition, or Q pattern, has not been validated experimentally in absorbing media.

The Pennes’ Bioheat Equation (PBHE) is commonly employed to model heat dissipation in biological tissues [24] where the heating term of the PBHE is the Q pattern of the FUS sonication. Finite-difference computational approaches to the PBHE are efficient enough to perform 3 D patient simulations in clinically relevant time frames, on the order of seconds to a few minutes and is widely incorporated for FUS applications [25,26].

A number of studies have compared experimental measurements of FUS metrics to simulations for validation. Raster or single-point hydrophone measurements of acoustic pressure after propagation through a homogeneous or heterogeneous medium have been used to show agreement between experimental and simulated acoustic pressures [27–30]. Thermocouple measurements, when corrected for viscous heating effects, have also been used to compare local experimental temperature rise to that from FUS simulations [27,28]. While data from hydrophone and thermocouple measurements are acquired at limited time points and spatial locations, MRI thermometry (MRTI) can provide highly sampled 4 D temperature measurements of experimental FUS sonications. 2 D MRTI has previously been used for validating FUS simulations in homogeneous phantoms [30] and *ex vivo* porcine muscle [21], although spatial averaging was cited as a source of error for measuring peak temperature rise. With appropriately high resolution, spatial averaging effects can be significantly decreased and MRTI can provide an efficient acquisition of 4 D temperature data for validation [31].

The aim of this study, therefore, is to validate HAS simulations in an independently characterized tissue-mimicking phantom model [32] using 4 D MRI temperature measurements. HAS acoustic predictions of MR-guided focused ultrasound (MRgFUS) sonications are combined with a previously validated PBHE thermal solver [25], allowing for quantitative comparison of simulated three-dimensional (3D) temperature profiles to the magnitude and shape of experimental 3 D MRTI profiles over time.

Accurate temperature predictions are dependent on the accuracy of the tissue property input parameters. Both acoustic and thermal simulation algorithms require *a priori* knowledge of several tissue-specific properties, which are difficult to measure *in situ*, usually requiring modelers to rely on published table values [7,9,10,33]. These values are often aggregated from multiple studies and different measurement techniques, and include both known and unknown degrees of uncertainty that affect the final simulated outcome. Even when samples are measured directly, variability in acoustic property values has been observed across laboratories [34]. Additionally, the field of FUS is progressing towards standards for consistently measuring transducer output power and beam profile [35]. In one study validating FUS metrology in phantoms, instrument uncertainties for measuring transducer output and phantom properties were quantified and simulations were designed to reveal the extreme over-and under-estimation of temperature profiles based on the uncertainties [28].

A secondary aim of this study is to perform an uncertainty analysis of the HAS and PBHE models using a Monte Carlo sampling approach. For experimental validation, model input parameters characterizing the transducer and gelatin properties are independently measured. Then, for uncertainty analysis, the parameter measurement uncertainties are propagated through the models to determine if the simulated temperature profiles fall within the expected uncertainty range of the model. Finally, a local sensitivity analysis is performed on simulation accuracy metrics to quantify the impact of the uncertainty of each tissue property on simulated temperature profiles. For validation and uncertainty analysis purposes, simple homogeneous phantoms were chosen to minimize variability in phantom construction and properties across trials.

## Methods

### Tissue-mimicking gelatin phantoms

Gelatin-based tissue-mimicking homogeneous phantoms were made with four different concentrations of evaporated milk to vary the acoustic and thermal properties, as previously described [32]. For this study, 11.1%/v of 250-bloom ballistics gelatin (Vyse Gelatin Co., Schiller Park, IL, USA) was dissolved in an evaporated milk (Nestl´e Carnation Evaporated Milk) and de-ionized water mixture. To vary the acoustic properties, the composition ratio of milk-to-water was varied at 10, 30, 50, or 70% milk by volume. The gelatin mixture was poured into cylindrical molds (3-cm radius, 7-cm height), which were sealed on both ends with thin PVC film that served as an acoustic-transparent window. All phantoms (*n* = 3 per composition type for a total of twelve phantoms) were constructed one week prior to experiments and stored at 4°C to ensure complete formation of the gelatin.

### Parameter measurements

To determine nominal values for parameter inputs and their uncertainties for the Monte Carlo statistical analysis, acoustic and thermal properties were measured in the gelatin phantoms by independent methods as described below. All gelatin properties were measured at room temperature (23 ± 0.75°C). All mean parameter values and the source of their uncertainties are summarized in Table 1.

### Acoustic Absorption

The acoustic pressure absorption coefficient (*α*_a_) was set equal to the total acoustic attenuation (*α*) in the gelatin phantoms by assuming negligible scattering effects [36]. Absorption was measured by two methods to verify the absence of systematic bias or offset. First, acoustic attenuation was measured with through-transmission ultrasound using the substitution method, as previously described [32]. Twelve hours prior to measurements, phantoms were removed from 4°C storage and brought to room temperature (~23°C). A single-element broadband transducer centered at 1 MHz (Panametrics-NDT, V314, Watham, MA, USA) and a receiver hydrophone (ONDA, HNR-0500, Sunnyvale, CA) were used to measure the loss of acoustic pressure of a five-cycle burst through the phantom sample with reference to water. Attenuation was measured at four central frequencies, 0.65, 1.05, 1.80, and 3.00 MHz, and a line fit to Equation (1) to determine the attenuation at 1.0 MHz

(1) $$\text{α}\;\text{=}\;{\text{α}}_{0}f^{c}$$

where α_0_ is the acoustic pressure attenuation at 1 MHz (Np/cm), *f* is the transducer frequency (MHz), and *c* is the power coefficient of the frequency dependence. A single measurement was made of each of the twelve phantoms. The measured frequency parameter *c* was not statistically different among the phantoms, with an average value of 1.00 ± 0.09 (*n* = 12); therefore, attenuation was assumed to be linear with frequency for all phantoms. A water-filled phantom of equivalent dimensions was used as the water reference.

Attenuation for two of the compositions was also measured by determining the insertion loss using a radiation force balance technique [37]. For these measurements, one additional phantom was poured from the batch mixture of the 30% and 50% milk composition phantoms. A 256-element phased-array transducer (Imasonic, Voray-sur-l’Ognon, France; 10-cm radius of curvature, 14.4 × 9.8 cm aperture) was used to focus ultrasound (*f* = 940 kHz, *P*_out_=10 electrical W,) through the sample into an acoustically absorbing brush (uniformly potted plastic bristles, 2.5 cm long) in the far field (3 cm distal to the geometric focus) suspended from a balance (±0.001 g precision). The radiation force on the brush was measured during 20-s sonications and absorbed power in Watts was calculated as

(2) $$P_{abs}=cgm/k$$

where *c* is the speed of sound in water (1500 m/s), *g* is the gravitational constant (9.8 m/s^2^), *m* is the average mass reading on the balance (kg) obtained over the 20-s time interval, and *k* is a correction factor to account for the average cosine of the cone of ray angles from the transducer elements to the brush (*k* = 0.919) [38]. The phantom samples, which had a sufficiently large diameter (11-cm diameter, 3-cm thickness) to encompass the entire focused beam, were placed in the near field of the focus and the sample pressure attenuation coefficient (Np/cm) was calculated as

(3) $$\text{α}\;\text{=}\;\text{In}\left(\frac{P_{s}}{P_{r}}\right)/2d_{e}$$

where *P*_*s*_ the measured power with the sample in place, *P*_*r*_ is the measured power with a water-filled phantom in place, and *d*_*e*_ is the effective path length (3.2 cm) o1f the beam through the sample, accounting for the average angular spread of the focused beam.

The average attenuation values as measured by through-transmission for each of the four phantom compositions (*n* = 3) were used as the mean values, while the differences between the through-transmission and radiation force balance measurements of the 30% and 50% phantoms were used to determine measurement uncertainty.

### Speed of Sound

The speed of sound of the phantoms was determined with the through-transmission method as described above. Using substitution [32], the time-of-flight difference between the sample and water reference was used to calculate the speed of sound of the sample. Parameter uncertainty was defined as the average standard deviation of experimental measurements (*n* = 3 per phantom).

### Acoustic Power

The power efficiency (acoustic power divided by applied electrical power) of the transducer was measured by radiation force balance in water at the applied nominal experimental electrical powers: 20 and 25 W. The measured transducer efficiency was constant (32%) over this range; therefore, changes in brush buoyancy as a result of local heating were assumed to be negligible. Because the reported electrical power output of the transducer generator (Image Guided Therapy, Pessac France) varied slightly between shots, the electrical power reported during the MRgFUS experiments was averaged within each of the two sonication powers (*n* = 12). Then, these averaged reported powers were multiplied by the measured transducer efficiency (32%) to obtain the average experimental acoustic power for each sonication level (6.3 and 7.9 W, respectively). Parameter uncertainty was determined by error propagation of the standard deviation of force balance measurements (*n* = 30 continuous 2-s FUS shots per power level) and standard deviation of the reported electrical output power of the transducer during experiments (*n* = 12 sonications).

### Thermal Properties

A KD2 Pro thermal probe (SH-1 attachment, Decagon Devices, Inc., Pullman, WA, USA) was used to measure the thermal conductivity (κ) and volumetric heat capacity (VHC) of the phantoms. The two-pronged probe was inserted into each phantom (*n* = 3 per milk/water ratio) to obtain the thermal properties. Finally, the density (*ρ*) of the phantoms was measured via the volume displacement method (*n* = 3 per milk/water ratio). The parameter uncertainty for κ and VHC was defined as the manufacturer reported error of the KD2 Pro probe for each property. The parameter uncertainty for density was defined as the average standard deviation of the experimental measurements (*n* = 3 per phantom).

### Experimental FUS Sonications

**Figure 1.**
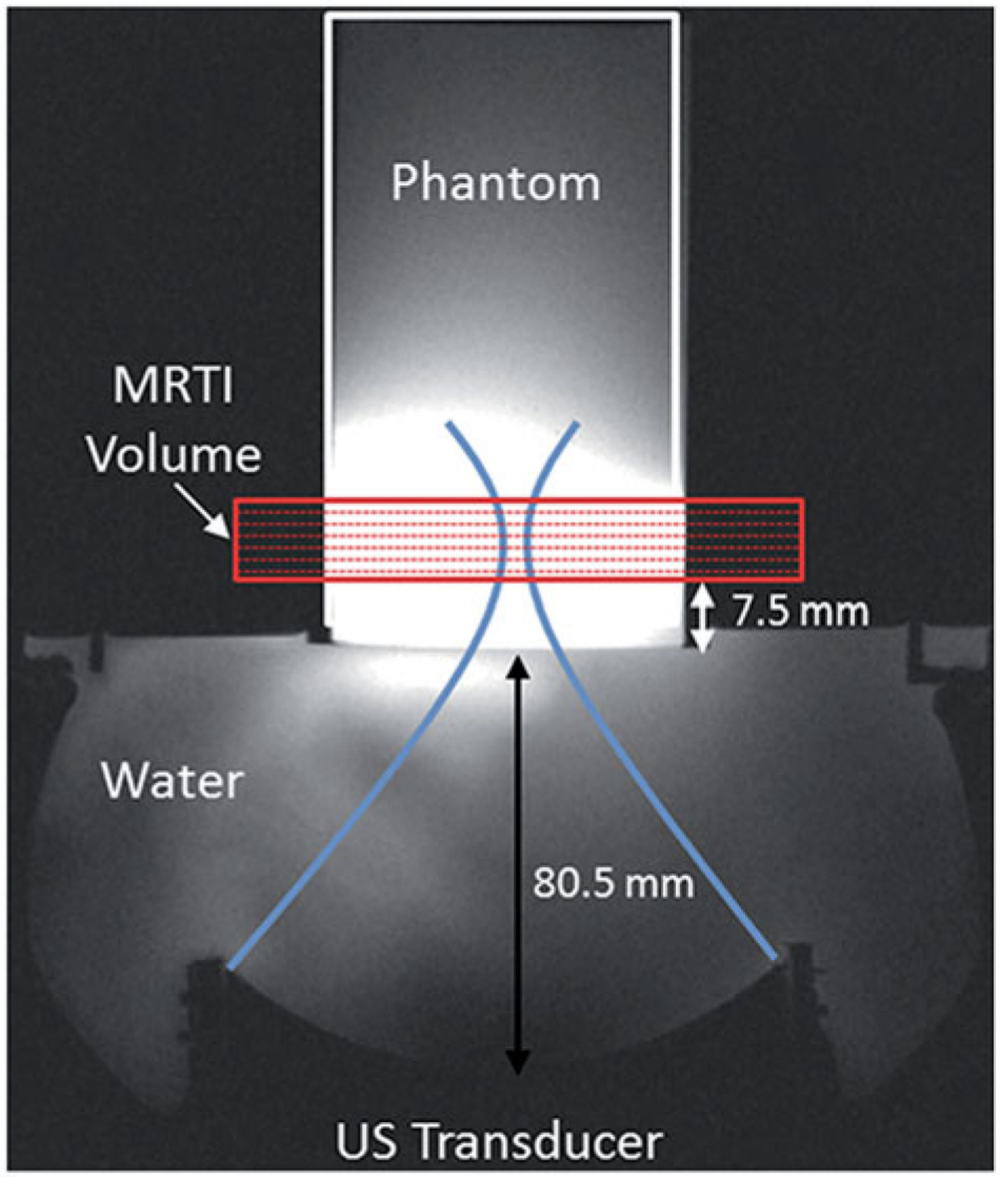
Experimental set-up for FUS sonications in homogeneous gelatin phantoms with real-time volumetric MRTI as shown in an axial T1-weighted MR image. (A) 256-element phased-array transducer is positioned below the phantom cylinder, coupled with room-temperature degassed, deionised water. The geometric focus was placed 19.5 mm into the base of the phantom. The MRTI image slab was oriented perpendicular to the direction of FUS beam propagation, with slices centred at the geometric focus. The base of the MRTI volume was 7.5 mm above the bottom of the phantom.

Sonications were performed with an MRgFUS system (Image Guided Therapy, Pessac, France) with a 256-element phased-array ultrasound transducer (Imasonic, Voray-sur-l’Ognon, France; 10-cm radius of curvature, 14.4 × 9.8 cm aperture) with an operating frequency of 940 kHz and a transverse focal size of 2.4 × 3.8 mm, (FWHM of pressure profile measured in water by hydrophone). Phantoms were removed from 4°C storage 12 h prior to experimentation to allow for thermal equilibrium at room temperature (mean =21.4°C). Samples were secured above the transducer such that the geometric focus was consistently 19.5 mm above the bottom of the phantom and coupled to the transducer with degassed, deionized water (Figure 1). Sonications were applied without steering at either 6.3 or 7.9 acoustic watts for 18.16 s. Ultrasound heating was synchronized to start 2.0 s after the start of MRTI acquisition (3 T Prisma^FIT^, Siemens) via optical triggering. The 6.3- and 7.9-W sonications were performed three times each (*n* = 3) in all four phantom compositions. Each sonication level was applied to opposite ends of the phantom to prevent thermal hysteresis effects caused by repeated heating.

MRTI was acquired using the proton-resonance frequency (PRF) method, which has been shown to respond linearly in aqueous tissues over FUS ablation temperature ranges [39]. A 3 D coronal volume centered at the geometric focus was acquired during 18.16 s of FUS heating and 26.9 s of cooling (TR/TE =28/12 ms, *t*_acq_=3.36 s, 1 × 1×3 mm resolution, 8 slices with 25% oversampling). Raw data were reconstructed in MATLAB and zero-fill interpolated to 0.5 mm isotropic. Temperature change was calculated from the change in the image phase, with a water proton chemical shift (WPCS) coefficient set to –0.01 ppm/°C, which is a commonly assumed value for aqueous tissues [40]. The signal-to-noise (SNR) of each MRTI volume was calculated as the peak temperature rise divided by the background noise, defined as the standard deviation in temperature over time in the non-heated phantom (36-voxel region). Temporal temperature drift was corrected in each MRTI volume by fitting a line to the temporal temperature curve in the same non-heated region. The linear correction (–0.007°C/s on average) was then applied voxel-wise to the respective MRTI volume. Finally, temperature data at each sonication level and phantom type were averaged (*n =* 3) to form the experimental data set.

### Simulation

#### Acoustic Simulation

The HAS method was implemented as previously described [23] to simulate the complex acoustic pressure and resulting power deposition pattern Q (W/m^3^) for each FUS sonication in the gelatin phantoms. To reduce computation time, an element response function array (ERFA) is generated one-time for the 256-element transducer prior to HAS simulation by using the Rayleigh-Sommerfeld integral to calculate the initial pressure from each element through the water interface to the front plane of the model according to their manufacturer-specified locations on the transducer face. At run-time, the assigned phase and amplitudes of each element are applied to the ERFA file, whose complex terms are summed to obtain the initial plane pressure pattern in water. The initial pressure plane in water is imposed onto the front face of the model; therefore, refraction and first-order reflections are calculated at the water-phantom interface proximal to the transducer and at each successive layer during propagation. Each voxel in the 3 D model of the homogeneous phantoms was assigned an acoustic attenuation coefficient (*α*), speed of sound (*c*), and density (*ρ*) (Table 1). For all simulations, water voxels were assigned the following property values: *α =* 0.00 Np/cm/MHz; *c =* 1,500 m/s; *ρ =* 1000 kg/m^3^. The 3 D model was generated by extruding a 2 D segmented coronal image of the phantom cross-section along the height of the phantom cylinder (70 mm). Additional inputs included the transducer geometry, frequency, power, and distance from the 3 D model, as summarized in Table 2. Simulations were run with 0.25-mm isotropic model resolution (model size =647 × 343 × 280 voxels).

#### Thermal Simulation

A previously validated 3 D finite-difference PBHE solver was implemented in MATLAB to simulate the 3 D temperature profiles [25]. Similar to the HAS simulations, each voxel of the 3 D phantom model was assigned a density (*ρ*), thermal conductivity (κ), and volumetric heat capacity (VHC) value (Table 1). The remaining inputs to the solver included the Q pattern obtained from HAS, the duration of FUS heating and the temporal resolution, as summarized in Table 2. Simulations were run at 0.25-mm isotropic resolution and the results were down-sampled by linear interpolation to match the spatial resolution of the experimental temperature profiles (0.5mm isotropic). Because temporal resolution of the simulation is higher than that of the MRTI, the simulated profiles were averaged over the time-span of each MRTI acquisition (∆*t* = 3.36 s).

### Quantitative validation of model

To validate the HAS approach for potential treatment-planning applications, the mean experimentally measured parameters (Table 1) were used as inputs for the simulations. Simulations were run for all phantom concentrations and acoustic power levels. The error between the spatially registered experimental and simulated temperature profiles was calculated as the experimental value subtracted from the simulated value. Four output metrics, *Y*_*i*_, were used to assess error: spatiotemporal peak temperature rise and location, and transverse and longitudinal full-width-half-maximum (FWHM) values. All error metrics, *E*_*i*_, were calculated as

(4) $$E_{i}=\frac{100*\left(Y_{i,sim}-Y_{i,\exp}\right)}{Y_{i,\exp}}$$

where *Y*_*i,*exp_ is calculated from the averaged experimental temperature profiles (*n* = 3).

### Monte carlo uncertainty analysis

A Monte Carlo (MC) statistical approach was implemented for uncertainty analysis of the simulations for the given parameter space. All experimentally measured input parameters, *X*_j_, were generated as normally distributed random variables (RVs) with respective standard deviations, ${\text{σ}}_{{\text{X}}_{j}}$. Acoustic attenuation, speed of sound, acoustic power, and density variables were modeled as independent normal distributions, where the population mean and standard deviation were the experimental mean and uncertainty of the independent measurements (Table 1). Thermal conductivity and volumetric heat capacity variables were modeled together as a normal joint distribution, where the population means and covariance matrix were derived from the experimental means and uncertainty of the KD2 Pro probe measurements (Table 1) [41]. A joint distribution was chosen for these thermal properties because they were not measured independently.

To determine the number of iterations *n* for the MC uncertainty analysis, the simulation was initially run 50 times varying all parameters according to the uncertainties listed in Table 1. Calculated from the outcome of this subsample, the ideal *n* was found using

(5) $$n={\left[1.96\frac{100\sigma_{sub}}{\varepsilon {\bar{Y}}_{sub}}\right]}^{2}$$

where 1:96 is the normal distribution z-score for a 95% confidence interval, σ_*sub*_ is the standard deviation of the model output $\left(Y\right),\;{\bar{Y}}_{sub}$ is the mean of the model output, and ε is the expected experimental variability defined by

(6) $$\text{ε}\;\text{=}\;\frac{100}{SNR}\sim 3\%$$

where the SNR of the MRTI data is calculated as described above. The average value of e in the experimental data was 3%, which is comparable to the expected error of clinical MRTI sequences given background noise of ±1°C and a 20°C temperature-rise for ablation (ε ≈ 5%) [42]. Using data from simulations in 30% milk phantoms at acoustic power of 7.9 W and (5), the value of *n* was determined to be 71. This calculated *n* value was assumed to be valid for MC simulations in all combinations of phantom types and power levels.

#### Local sensitivity analysis

Two methods of local sensitivity analysis were implemented to provide an estimate of relative sensitivity of input parameters on the HAS output. First, a least-squares linear regression was fit to an output metric of the MC simulations (*n* = 71 × 8 = 568). Given a sample matrix consisting of *k* input variables, *X*_*j*_, where each iteration *n* results in an output metric, *Y*_*i*_, the matrix form of the linear regression is

(7) $$Y_{i}=\;b_{o}+\sum_{j=1}^{k}b_{j}X_{ij}$$

where *b*_*o*_ and *b*_*j*_ are the intercept and regression coefficient, respectively. The standardized regression coefficient (SRC) is then calculated by

(8) $$SRC_{j}=b_{j}{\text{σ}}_{X_{j}}/{\text{σ}}_{Y}$$

where ${\text{σ}}_{{\text{X}}_{j}}$ is the standard deviation of *X*_*j*_ and σ_*Y*_ is the standard deviation of the output metric *Y* [43]. Since the *R*^2^ value of the regression is close to 1 for all output metrics (Table 4), this linear regression analysis is appropriate for the HAS and PBHE models used in this study.

Additionally, the contribution of each input parameter’s uncertainty on the overall model uncertainty was assessed in the local parameter space. All but one parameter at a time were held constant at the experimental mean value for *n* = 71 MC iterations. The uncertainty, *U*_j_, of the output metric resulting from each parameter was calculated as

(9) $$U_{j}=\frac{100*{\text{σ}}_{{\text{X}}_{j}}}{{\bar{Y}}_{j}}$$

where ${\bar{Y}}_{j}$ is the mean of the output when variable *X*_j_ is held constant. The relative uncertainty for each variable was also calculated as $U_{j}/{\text{σ}}_{{\text{X}}_{j}}$.

## Results

### Validation of HAS simulations with experimental temperature profiles

**Figure 2.**
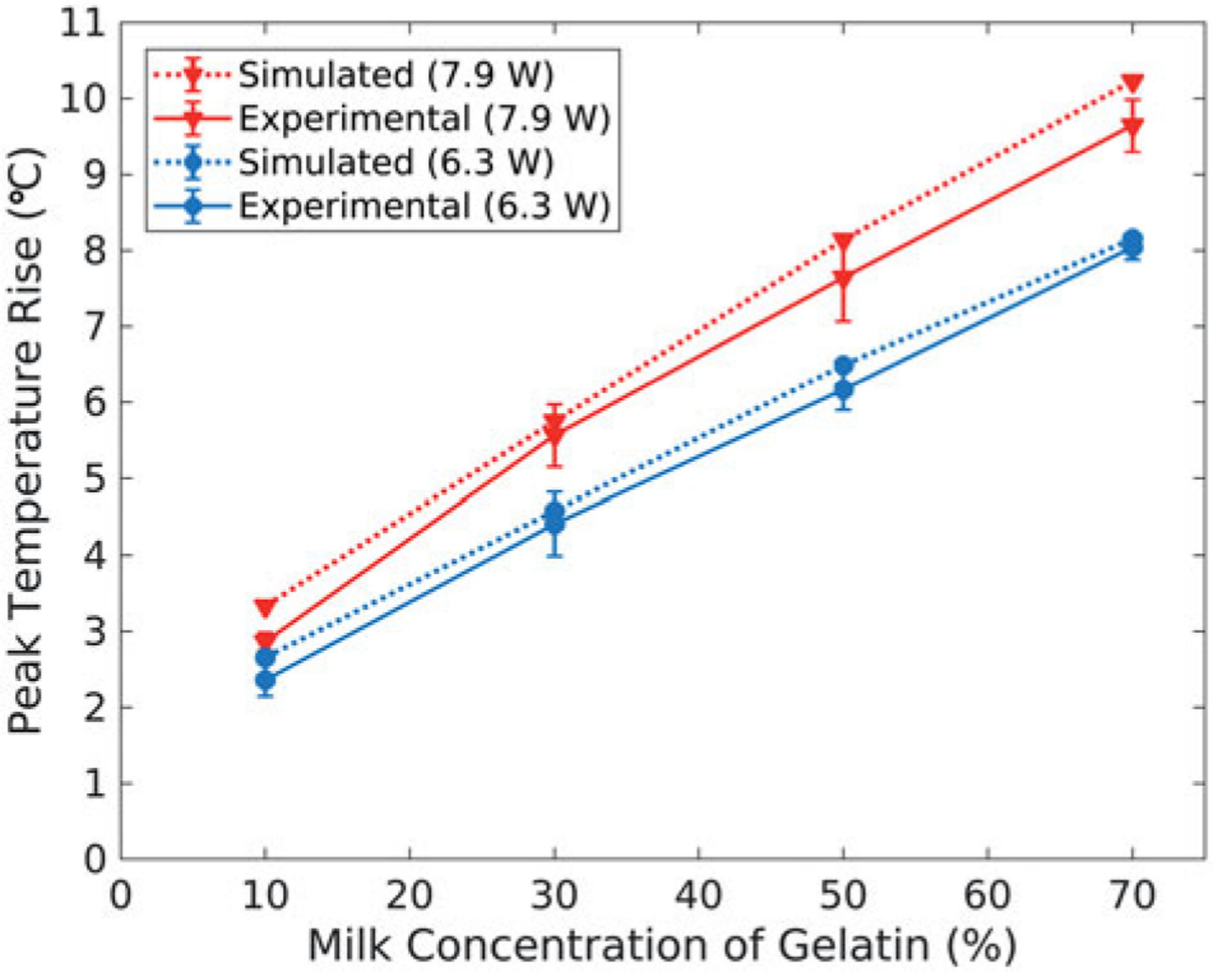
Average peak temperature rise achieved in the simulated (dashed, *n* 1) and experimental (solid, *n =* 3) temperature profiles, with experimental error bars representing one standard deviation. Results are plotted as a function of milk concentration of the gelatin phantoms for both FUS sonication powers: 6.3 W (blue, circles) and 7.9 W (red, triangles).

Table 3 summarizes the spatial and temporal results comparing experimental MRTI data to the HAS and PBHE thermal simulations. The spatiotemporal peak temperature rises of the experimental and simulated sonications are plotted in Figure 2. The peak temperature rise in the experimental sonications ranges from 2.4–9.6°C. The simulated temperature profiles over-estimates the mean experimental peak temperature rises (*n* = 3) by +0.33 ± 0.17°C, or +6.9 ± 5.0%, on average across all sonications. The low-temperature rise in the 10% phantoms results in a much higher percent error compared to the same temperature over-estimation in the other compositions (+0.38°C or +13.6% error). Finally, the simulated and experimental temperature rise results increase linearly with milk concentration at similar rates (0.092 and 0.091°C/% milk at 6.3 W; 0.115 and 0.102°C/% milk at 7.9 W, respectively).

**Figure 3.**
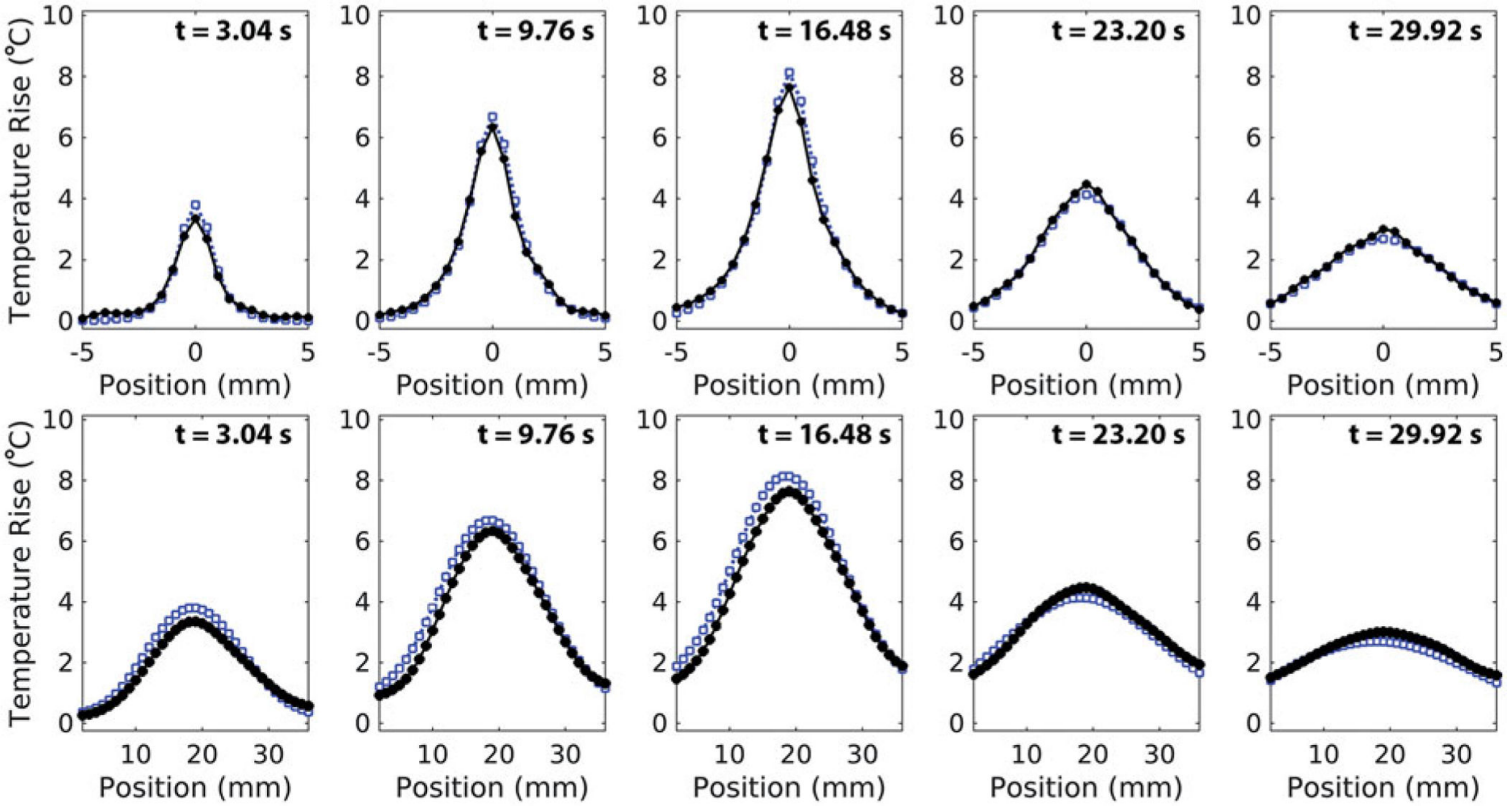
Transverse (top) and longitudinal (bottom) profiles of mean experimental (circle, black) and simulated (square, blue) temperature data in 50% milk composition phantoms at 7.9 W. Depicted transverse profiles are along the short-axis of the transducer face. For longitudinal profiles, the transducer is located to the left of the profiles. Experimental and simulated profiles are compared at multiple times points during heating (*t =* 3.04 and *t =* 9.76 s), at the time of experimental peak temperature rise (*t =* 16.48 s), and during cooling (*t =* 23.20 s and *t =* 29.92 s). Simulated profile resolution is down-sampled to 0.5 mm isotropic to match experimental spatial resolution.

Representative transverse and longitudinal temperature profiles at various time points are shown in Figure 3. Throughout heating and cooling, the simulated profile shape and width closely matches those of the experimental profiles. At the time of peak temperature rise, the error in the simulated transverse and longitudinal FHWM is –0.042 ± 0.04 mm (–1.9 ± 1.5%) and +1.37 ± 0.50 mm (+7.45 ± 3.0%), respectively. Finally, across all sonications, the simulated profile centre is consistently shifted towards the transducer by 0.94 ± 0.18 mm relative to the experimental profile centre.

**Figure 4.**
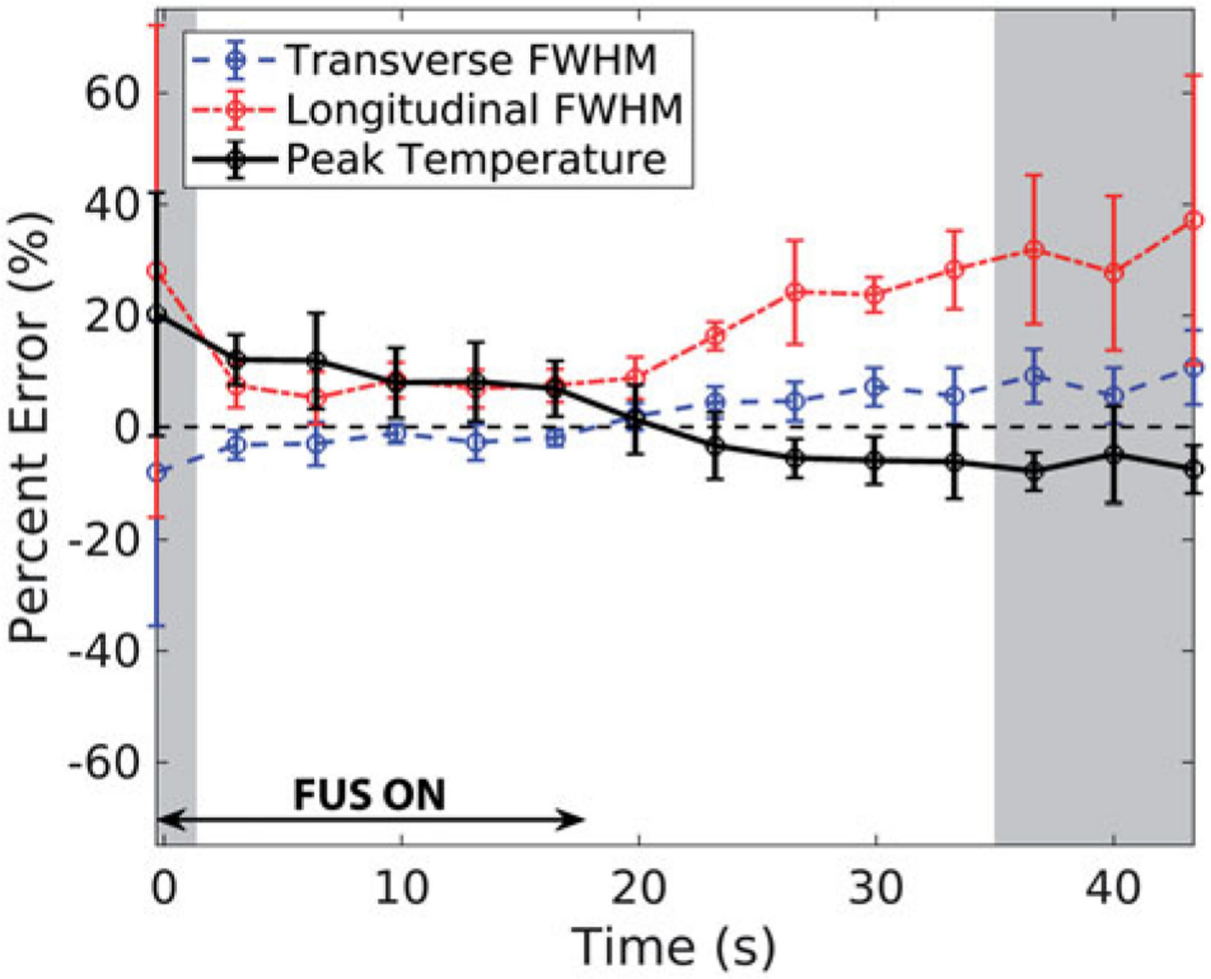
Average percent error in spatio-temporal peak temperature rise (black), transverse (blue, dashed) FWHM, and longitudinal (red, dotted) FWHM for all phantoms and sonication powers is plotted throughout FUS heating and cooling (*n =* 8; error bars ± one standard deviation). The shaded regions represent time-points during which the average spatial SNR in the MRTI experimental data across all phantoms was ≤ 20.

The error in simulated FWHM and peak temperature rise is plotted throughout FUS heating and cooling in Figure 4 The error bars represent the standard deviation in error values across all phantom types and power levels (*n* = 8). In general, error and the variability in error increase when SNR is below 20 (shaded time-points), which is the assumed SNR achieved in clinical ablations as explained in the Discussion. While the errors in transverse FWHM and peak temperature rise hover within a constant range over time, they invert between heating and cooling. Finally, although longitudinal FWHM is over-estimated in simulations throughout heating and heating, the error magnitudes follow the same trend as in the transverse direction.

**Figure 5.**
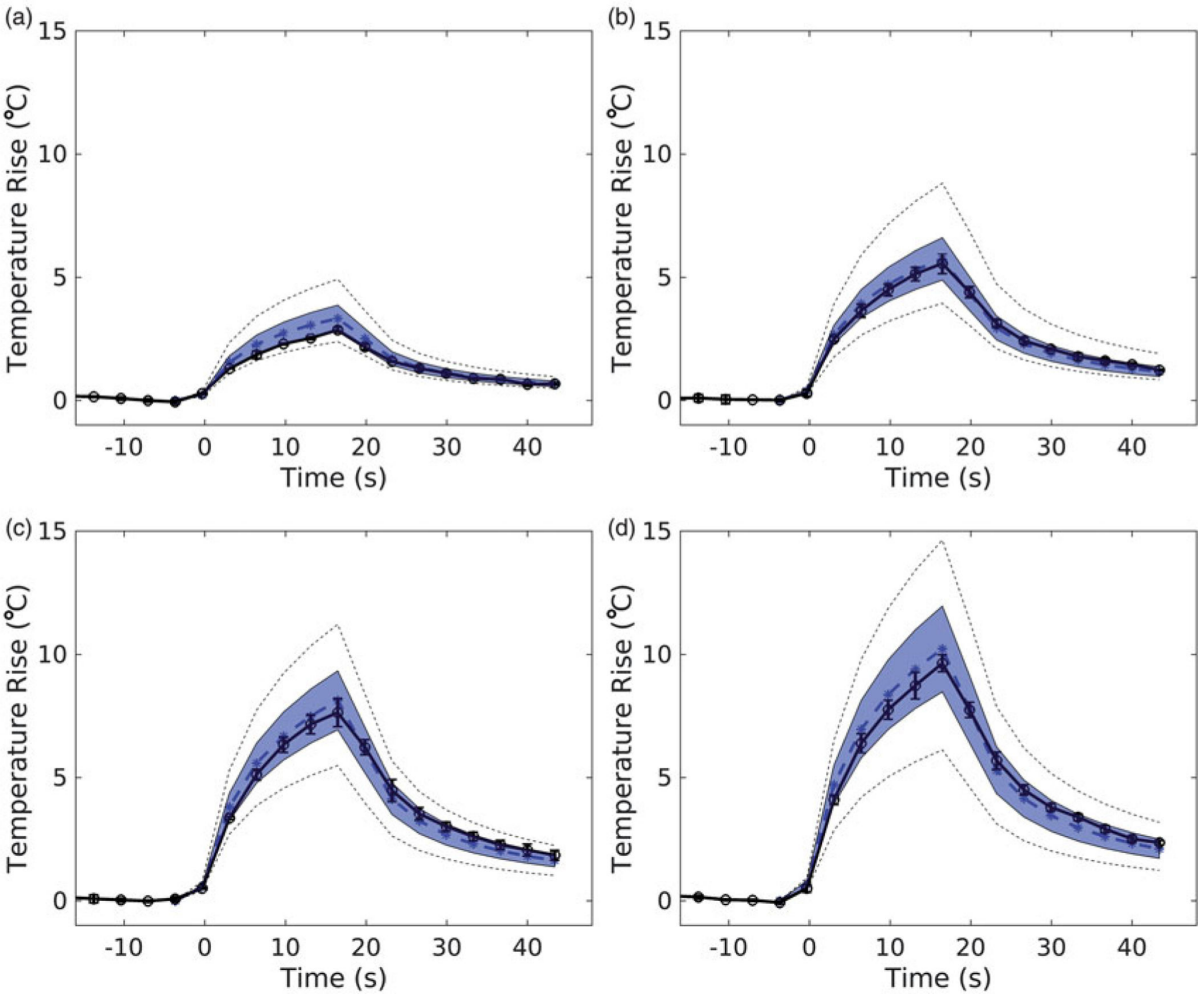
Temporal temperature curves of the peak temperature voxel from experimental (black, solid) and corresponding simulated (blue, dotted) temperatures for (a) 10%, (b) 30%, (c) 50%, and (d) 70% milk at 7.9 W sonication. Experimental error bars represent one standard deviation (*n =* 3). The shaded envelope represents one standard deviation of the MC analysis. The black dotted lines represent the high and low extremes of the MC analysis.

The results of the MC uncertainty analysis on the simulation output are visualized in Figure 5 for the 7.9 W sonications. The simulated peak temperature is plotted over time (blue, dotted; *n* = 1), with the shaded envelope representing one MC standard deviation of the same voxel over time (*n* = 71). The mean experimental peak temperature (black, *n* = 3), falls within the shaded region of uncertainty for all phantoms and powers. The average uncertainty, *U*, of the peak temperature in the MC simulations for all sonications is 00B114.6%. Uncertainties of the longitudinal and transverse FWHM are ±2.7% and ±1.3%, respectively.

### Local sensitivity analysis

Table 4 summarizes the results of linear regression analysis applied to the combined data set (*n* = 568) for three output metrics: spatiotemporal peak temperature rise, transverse FWHM, and longitudinal FWHM. The *R*^2^ value for all metrics was close to 1, indicating that the model is approximately linear in the temperature range and parameter space explored. Acoustic attenuation and acoustic power had the highest absolute SRCs with peak temperature as the output metric, followed by thermal conductivity, with speed of sound, density, and VHC having very low SRCs. Both FWHM metrics are most sensitive to thermal conductivity, followed by volumetric heat capacity, which have opposing correlations to the FWHM of the profile. Longitudinal FWHM was more sensitive to acoustic attenuation and speed of sound than transverse FWHM, while acoustic power had negligible effects on overall profile shape.

**Figure 6.**
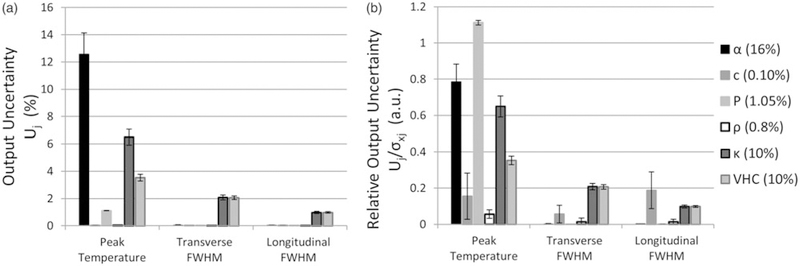
Output uncertainty (*U*_j_) and relative output uncertainty $\left(U_{j}{\text{σ}}_{{\text{X}}_{j}}\right)$ for three different output metrics of the MC simulations. (a) *U*_j_ when each parameter is varied individually (as specified in the legend). (b) $U_{j}{\text{σ}}_{{\text{X}}_{j}}$ comparing the relative impact of each parameter on U_*j*_. Output uncertainties were averaged over all phantoms and power levels. Error bars represent one standard deviation.

The quantified impact of each parameter’s measurement uncertainty on simulation uncertainty is depicted in Figure 6(a). Unsurprisingly, the parameters with the greatest uncertainty result had the greatest uncertainty across output metrics. However, Figure 6(b) depicts the non-proportional impact of each parameter on the model, which shows that their relative uncertainties were below 1, except in the case of acoustic power. For example, while acoustic power and speed of sound had minimal effects on output variability (Figure 6(a)), their effect relative to their input uncertainties was notable (Figure 6(b)). Finally, while peak temperature rise accuracy is sensitive to nearly all properties, the profile shape is sensitive only to thermal properties and speed of sound.

## Discussion

### HAS validation

Acoustic simulation methods are not easily validated against *in situ* experimental data involving an absorbing medium [13]; often novel methods are compared against existing models for determining accuracy [18,44]. Experimental validation requires a highly controlled environment with known input parameters, which can be difficult to achieve for FUS, particularly for *in situ* conditions [29]. This study sought to create such an environment with homogenous tissue-mimicking phantoms [32]. Additionally, by incorporating uncertainty measurements of properties known to have both intra- and inter-subject variability, the findings of the MC sampling analysis are clinically relevant.

This study demonstrates that when using directly measured simulation parameters, the HAS and PBHE models can adequately predict FUS heating in a homogeneous and non-perfused media as assessed by predicted focus locations, achieved temperature rises, and FWHM values. Additionally, experimental temperature plots fell within one standard deviation of the MC simulations (Figure 5). The errors between simulation and experiment are similar in scale to other FUS validations studies. When comparing measurements in homogeneous phantoms to the nonlinear KZK and PBHE simulations, Maruvada et al. reported simulated temperatures over-estimated the peak temperature by 1–20% and transverse FWHM error was less than 3% [28]. Haddadi et al. reported peak temperatures simulated by Westervelt equations were within 4.3–12.8% of measured values in *ex vivo* liver tissue [45]. When comparing simulated temperature profiles to those measured with 2 D MRTI in *ex vivo* porcine muscle, Solovchuk et al. found that 45°C temperature rises were over-estimated by 9–24°C when spatial averaging errors in the 2 × 2×8 mm voxels were unaccounted for [21].

The evolution of average profile errors over time shown in Figure 4 provides further insight into simulation errors. Simulated longitudinal FWHM was consistently broader throughout heating and cooling. When compared to 2 D hydrophone scans in water, the HAS simulated longitudinal pressure profile was 3.8% broader than the hydrophone measurement. A wider Q-pattern corresponds to lower temperature gradients in the PBHE simulation, which further broaden the temperature profile. The cause of the longitudinal FWHM discrepancy in the pressure profiles should be further explored. Although the surface velocity of each transducer element was assumed to be uniform in simulations, measurements with acoustic holography in water could reveal a non-uniform distribution [46]. The collective pressure pattern from non-uniform sources may contribute to error between experimental and simulated beam width in water. In phantom simulations, the errors in FWHM remained relatively stable during US heating, but increased after the ultrasound was turned off; therefore thermal property inaccuracies are the likely cause of discrepancies between simulation and experiment during cooling. Specifically, the input value for thermal diffusivity, which is the ratio of thermal conductivity to specific heat capacity, was likely higher than the true phantom value.

However, a high input value for thermal diffusivity does not explain consistent over-estimation of peak temperature during ultrasound heating. Alternatively, errors in the acoustic attenuation or power may have contributed to consistent over-estimation of the simulated peak temperature during ultrasound heating. Additionally, acoustic scattering was assumed to be negligible in the gelatin so the measured attenuation coefficient was attributed all to absorption. If scattering was present in the phantoms, the acoustic absorption implemented in HAS would be an over-estimation. Finally, the small difference in gelatin temperature during property measurements and during the FUS sonications (23.0°C and 21.4°C, respectively) as well as local temperature changes of the gelatin throughout heating are possible sources of error between simulated and experimental profiles. Local temperatures can cause inhomogeneities in property values over time and throughout the spatial temperature profile. Notably, the acoustic attenuation has been shown to decrease [47] and specific heat capacity has been shown to increase [48] with increasing temperature in gelatin. The combined effect of these dynamic properties on local heating may account for over-estimated simulated peak temperatures. In tissues, several acoustic and thermal properties are temperature-dependent in ablative temperature ranges [33, 49,50]. Although it is beyond the scope of this study, a few studies have aimed to incorporate thermally dynamic tissue properties into simulations [10,12].

Throughout heating and cooling, another possible source of error is the WPCS coefficient used for the MRTI calculation, –0.010 ppm/°C. In other water-based gels, this coefficient has been measured to be between –0.0085 and –0.0123 ppm/°C [51–54], or –15 and +23% of the assumed value in this study. A decrease in the actual WPCS coefficient would proportionally increase the experimental temperature values, and vice versa. Additionally, although spatial averaging effects are reduced in comparison to previous MRTI validation studies [21,30], simulated data were down-sampled, not spatially averaged to match MRTI resolution. Therefore, MRTI measurements could be slightly underestimated. Finally, poor SNR had a moderate effect on experimental data variability and the resulting simulated error calculations. The level of noise in clinical PRF MRTI scans has been reported to be on the level of ±1°C [42], which for a 20°C temperature rise would result in an SNR of 20.0. The shaded regions in Figure 4 denote time-points where the average SNR across all phantoms and power levels was ≤20. The errors are more variable and less smoothly varying during these time-points at the start of heating and end of cooling. In phantoms with 2–5.5°C temperature rises, the SNR was below 20 for the majority of cooling.

At the peak temperature time-point, the normalized transverse and longitudinal profiles (Figure 5) closely match in profile shape and width; however, simulated longitudinal profiles were consistently shifted towards the transducer by ~0.94 mm. Focal depth is dependent on transducer geometry and the refractive index at the water-phantom interface, which is modeled in the HAS algorithm. Because the speed of sound of the phantoms is similar to that of water and there was low variability in the speed of sound measurements in phantoms (±0.1%), it is unlikely that an error in this property measurement is causing the focal shift. However, the consistent 0.94 mm focal shift is within the manufacturer-reported tolerance of the transducer focal length (± 3 mm) and the in-plane resolution of the MR T1w images (1 × 1 mm) used to calculate the transducer distance from the phantom in the experimental sonications.

Absolute errors in peak temperature and transverse and longitudinal FWHM metrics were not correlated with experimental temperature rise (Pearson’s *r* = 0.2, –0.1, and –0.04, respectively); therefore, the absolute error is not expected to increase at higher temperatures if propagation remains linear and assuming that acoustic and thermal properties are accurately measured. At higher temperatures and penetration depths, it is expected by deduction that errors in tissue properties would result in higher absolute simulation errors.

Although this validation study was performed in homogeneous phantoms, HAS and PBHE simulations in heterogeneous breast phantoms were also shown to correlate well with experimentally obtained MRTI data in a phase aberration correction study, although the quantitative comparison was not as rigorous as presented in this study [55]. Despite careful independent measurements of simulation parameters, this study demonstrates the challenges of validating FUS simulations in a fully characterized and controlled experimental setting.

### MC sensitivity analysis

MC sensitivity analysis of the HAS and PBHE simulations revealed that uncertainty in tissue properties and model parameters have a greater impact on the profile amplitude than spatial extent of the profile. As expected, the simulated peak temperature is highly sensitive to the accuracy of acoustic absorption and transducer power and moderately sensitive to tissue thermal properties. However, the average FWHM error between simulated and experimental profiles (–1.9 and +7.45%) was on the order of or greater than the MC simulation uncertainty (1.3–2.7%), indicating that the HAS and PBHE models had a greater impact on the simulated FWHM error than the uncertainty of the simulation parameters.

Local sensitivity analysis provides insights into which tissue properties require accurate measurement for treatment planning of FUS ablations. For example, density had a negligible relative impact on simulation uncertainty; therefore, density table values are likely sufficient for treatment planning purposes. Similarly, tissue speed of sound results in a low relative uncertainty on peak temperature rise (Figure 6(b)); however others have shown that speed of sound inaccuracies can affect phase aberration correction and accurate beam focusing [56]. Figure 6(b) confirms that peak temperature is directly proportional to acoustic power; thus it is important that transducer power efficiency is adequately characterized. Finally, thermal conductivity and volumetric heat capacity contribute moderately to the uncertainty of each temperature profile metric.

Of all tissue properties, acoustic attenuation uncertainty had the greatest impact on the simulated peak temperature uncertainty [28]. An accurate measurement of acoustic attenuation is difficult to achieve as evidenced by the discrepancy between the through-transmission and radiation force balance measures implemented in this study (16%) as well as the large uncertainty of literature-reported values in *ex vivo* tissues [55]. Within this study, differences in the attenuation measurement methods may have contributed to the observed discrepancy. For example, interface reflections were estimated and incorporated in the attenuation coefficient calculation in the through-transmission method, but not in the radiation force balance method. Given that the greatest impedance mismatch, that of water and 70% phantom, results in only a 0.05 pressure reflection coefficient assuming normal incidence (intensity reflection coefficient equal to *R*^2^=0.0025), differences in reflection and diffraction was likely not the main source of error between the two measurement techniques. Another possible source of error between the techniques may have been differences in room temperature, thus water and phantom temperature, during measurements (±0.75°C). Detailed investigations of each technique’s sources of error have been the subject of other studies [58,59] and should be considered when acquiring *ex vivo* acoustic property measurements.

The uncertainties used for each parameter of the MC analysis are similar to the standard deviation seen in published values of commonly targeted tissues such as muscle, prostate, brain, glandular breast tissue, fat, and bone. According to the IT’IS Foundation’s compiled literature values for these tissues, density, heat capacity, thermal conductivity, and speed of sound have within-tissue standard deviations of 4.5%, 10.9%, 7.2%, and 3.2%, respectively [60]. Previously published literature values for acoustic attenuation in these tissue types varied by up to 10% in these tissue types [57]. This sensitivity analysis provided an approximate quantification of expected model uncertainties given the range of values currently provided in the literature. For example, an uncertainty of 10% in acoustic attenuation values would result in a ~8% uncertainty in the expected peak temperature rise using the models presented in this paper, based on Figure 5(b). For a 20°C temperature rise, the peak temperature uncertainty would be ±1.6°C.

The sensitivity analysis of this model bolsters the need for accurate patient-specific tissue properties when modeling FUS ablation treatments. To address variability in table values of properties, variability in patient-specific properties, and temperature-dependent tissue properties, a few studies have proposed methods for estimating acoustic attenuation *in vivo* for treatment-planning purposes [61–63]. Similarly, methods for estimating thermal tissue properties *in vivo* have been explored [64–66].

### Limitations

A limitation of this study is the moderate temperature range over which the models were validated. The FUS-induced temperature rises in this study remained below 10°C to prevent temperature-dependent property changes or melting of the gelatin phantoms, but clinical ablations require temperature rises in the range of 20–35°C in order to achieve tissue necrosis. Although the results did not indicate a trend, the expected uncertainty in simulation results could increase with significantly higher temperatures (90–100°C in tissues) or pressures with the introduction of boiling or non-linear effects. Additionally, inherent to the HAS algorithm is the calculation of the initial pressure plane in water using the Rayleigh-Sommerfeld (RS) integral, which has been previously validated for weakly focused ultrasound sources [67]. Although the RS propagation method is commonly implemented for modeling focused sources, it is only valid for small to moderate apertures, and at a large enough distance from the transducer face [68]. Errors in the initial pressure plane are expected to be small based on these criteria but would also contribute to FWHM and pressure amplitude errors. Finally, despite the speed and convenience of the HAS method, studies have shown that for some types of FUS ablations, non-linear acoustic effects are not negligible [21]. Therefore the use of this model for treatment planning will need to be considered based on the intended application.

## Conclusion

The accuracy of the HAS acoustic modeling algorithm coupled with the PBHE thermal simulation for predicting FUS-induced temperature profiles has been quantitatively validated in homogenous gelatin phantoms over a range of acoustic properties. Simulated spatiotemporal peak temperatures and transverse and longitudinal FWHM profiles were in good agreement with experimental profiles measured with MRTI. HAS facilitates computationally fast (orders of seconds to minutes) and accurate simulations of linear propagating FUS sonications and the resulting temperature profiles.

In general, the peak temperature rise accuracy is highly sensitive to the acoustic power and attenuation used in the HAS model. The PBHE model and thermal properties have a secondary effect on simulated peak temperatures. The speed of sound, thermal conductivity, and volumetric heat capacity were the only properties to affect temperature profile width. However, the resulting uncertainty of FWHM values was small compared to the FWHM error in simulated versus experimental temperature profiles.

Future work for validating HAS treatment planning will include using non-invasive methods for estimating *in vivo* tissue properties as model inputs, as well as implementing heterogeneous treatment targets in both *ex vivo* and *in vivo* environments. HAS is currently being modified for inclusion of scattering and non-linear acoustic propagation effects and needs to be further validated under these conditions.

## Figures and Tables

**Table 1.** Parameters for acoustic and thermal simulations and their uncertainties for Monte Carlo analysis.

Simulation parameter	Symbol	Phantom type (% milk)	Mean (µ)	Measurement uncertainty $\left({\text{σ}}_{{\text{X}}_{j}}\right)$	Uncertainty determination
Acoustic power (W)	P		6.3	1.05%	Error propagation of force balance-measured efficiency and reported experimental transducer electrical output
			7.9		
Acoustic attenuation (Np/cm @ 1 MHz)	α	10%	0.015	16%	Difference between through-transmission and radiation force balance measurements (*n* = 3)
		30%	0.027		
		50%	0.042		
		70%	0.053		
Speed of sound (m/s)	c	10%	1540.0	0.10%	Standard deviation of experimental measurement
		30%	1552.0		
		50%	1560.3		
		70%	1571.7		
Density (kg/m^3^)	ρ	10%	1022	0.8%	Standard deviation of experimental measurement
		30%	1050		
		50%	1040		
		70%	1050		
Volumetric heat capacity (kJ/m^3^·°C)	VHC	10%	3404	10%	Manufacturer reported error of measurement device
		30%	3361		
		50%	3371		
		70%	3381		
Thermal conductivity (W/m·°C)	κ	10%	0.554	10%	Manufacturer reported error of measurement device
		30%	0.545		
		50%	0.565		
		70%	0.525		

**Table 2.** Fixed acoustic and thermal simulation parameters and simulation computation time.

Acoustic (HAS)		Thermal (PBHE)	
Transducer frequency	940 kHz	Time step	0.08 s
Focus depth	19.5 mm	Heating time	18.16s
		Perfusion term	0kg/(m^3^·s)
		Boundary condition	Constant temperature
Model size	647 × 343 × 280	Model size	647 × 343 × 280
Model resolution (isotropic)	0.25 mm	Model resolution (isotropic)	0.25 mm
Average computation time	32.1 s	Average computation time	35.3 s

**Table 3.** Comparison of experimental (*n* = 3) and simulated (*n* = 1) temperature profiles at temporal peak of HIFU heating.

	Spatial peak temperature rise (°C)		Transverse profile FWHM (mm)		Longitudinal profile FWHM (mm)		MRTI Data (°C)	
Milk composition	Experimental	Simulation	Experimental	Simulation	Experimental	Simulation	Average noise^a^	Average SNR^b^
6.3 W								
10%	2.36 ± 0.21	2.66	2.73 ± 0.08	2.76	16.74 ± 0.81	19.00	0.240	9.84
30%	4.41 ± 0.42	4.58	2.88 ± 0.09	2.76	18.14 ± 0.33	19.00	0.156	24.69
50%	6.17 ± 0.26	6.49	2.77 ± 0.1	2.78	16.92 ± 0.46	19.08	0.160	32.39
70%	8.04 ± 0.16	8.15	2.78 ± 0.05	2.74	17.23 ± 0.50	18.92	0.201	40.13
7.9 W								
10%	2.87 ± 0.12	3.33	2.77 ± 0.19	2.76	17.52 ± 0.12	19.00	0.177	13.01
30%	5.57 ± 0.41	5.75	2.83 ± 0.12	2.76	16.84 ± 0.79	19.00	0.186	27.31
50%	7.64 ± 0.57	8.14	2.79 ± 0.12	2.78	17.88 ± 0.94	19.08	0.172	36.15
70%	9.64 ± 0.34	10.23	2.82 ± 0.06	2.74	17.39 ± 0.43	18.92	0.151	54.32
Average % error (E_i_)	+6.9 ± 5.0%		–1.9 ± 1.5%		+7.45 ± 3.0%			

a The standard deviation in time of a 36-voxel non-heated region in the phantom (*n* = 3).

b Experimental Peak Temperature Rise/Standard Deviation (*n* = 3).

**Table 4.** Linear regression analysis of all MC Simulations (*n* = 568).

		Peak temp rise		Transverse FWHM		Longitudinal FWHM	
		*R*^2^	SRC^a^	*R*^2^	SRC	* R*^2^	SRC
		0.979		0.991		0.991
Acoustic attenuation	α		0.825		0.024		0.078
Speed of sound	c		0.053		–0.002		–0.078
Acoustic power	P		0.275		0.001		0.001
Density	ρ		0.040		–0.004		0.018
Thermal conductivity	κ		–0.158		0.787		0.787
Volumetric heat capacity	VHC		–0.074		–0.734		–0.728

a SRC: Standardized Regression Coefficient.
